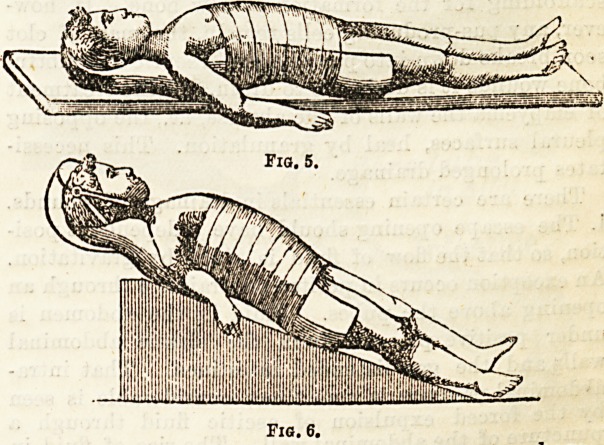# A Plaster Bed in the Treatment of Pott's Disease

**Published:** 1894-01-20

**Authors:** 


### MEDICAL PROGRESS AND HOSPITAL CLINICS.

[*The Editor will be glad to receive offers of co-operation and contributions from members of the profession. All letters should be addressed to* THE EDITOR, THE LODGE, PORCHESTER SQUARE, LONDON, W.]

### A PLASTER BED IN THE TREATMENT OF POTT'S DISEASE.

Dr. P. Redard, in a paper published in the *Journal de Medecine de Paris,* recommends the use of a plaster bed or shell in place of the well-known jacket ordinarily used, and a couch, on which the patient is placed, to restore the normal curvatures of the back. This bed, which he compares to the shell of a tortoise (carapace), fits exactly to the back of the head, the body, or the part of the limbs as the case may require, and is fitted while the patient is prone on a couch.

Dr. Redard does not descend to much detail, and we

FIG. 1.

FIG. 2.

FIG. 3.

are unable to fully understand the precise meaning of Figs. 1 and 2. In Fig. 3 we have a representation of a couch with adjustable pieces for the application of the plaster bed. On this the patient is placed, as in Fig. 4, proceeding with caution to allow the contracted muscles to relax, so that the abdomen of the patient may come to rest on the supports, and the easiest position obtained before the plaster is applied. When this has been done the back is covered with wool, over which is stretched calico to prevent the plaster from sticking. Bandages, impregnated with plaster and dipped in warm water, are then placed on in the following manner: One layer is put over the vertebral column from the neck or head as required to as low as is necessary; another starting from the same place passes diagonally to the side of the pelvis; while a third passes down the side of the body. We have no doubt more plaster is laid on to fill up the interspaces, but Dr. Redard makes no mention of this. The bed is then dried, varnished, trimmed, lined, and fixed in position by transverse bandages, as in Fig. 5.

In disease of the cervical region a jury mast of the usual pattern is used. (Fig. 6.)

With an apparatus so constructed we are told excellent results have been obtained, the advantages being the rigidity of the structure, its simplicity, its small cost, and the opportunities it gives of allowing the patient to get fresh air. It arrests the formation of deformity, relieves the muscular contractions, places the vertebræ in a favourable position for recovery, considerably relieves the pain, and the formation of

FIG. 4.

FIG. 5.

FIG. 6.

abscesses is prevented. In cases where abscesses have already formed, with the assistance of injections of iodoform oil and other simple surgical methods, recovery has rapidly taken place; and the same has been observed when the case is complicated with paraplegia. At no period of the disease is it too late to obtain relief by the use of this apparatus, and it makes no difference where the seat of the disease is. Dr. Redard recommends that the upright position should not be assumed until all pain has ceased and inflammatory signs disappeared.

The points about the use of this apparatus which appear to us of greatest value are that it can be readily removed to allow of ablutions—so necessary among the class of patients who make use of our hospitals—and the attempt that is made to restore the normal curvature of the vetebral column by the couch with adjustable parts. The triangle of Sayre makes no attempt to restore this beyond what is effected by the weight of the body. But here we have an apparatus with which we can, with a little patience and care, undo almost any amount of angular curvature that has arisen, and when this is effected an exact plaster cast can be taken, into which the patient is fixed and kept until the pathological processes have ceased. We think that Dr. Redard's plaster shell or bed is well worth a trial, and we confidently believe that surgeons will find it a useful method in the treatment of Pott's disease.

We are not so rash as to accuse a Frenchman of not being able to write his own language, but we feel obliged to say that Dr. Redard might have made his meaning a little clearer by better punctuation and re-arrangement of some of the sentences.

## Figures and Tables

**Fig. 1. f1:**
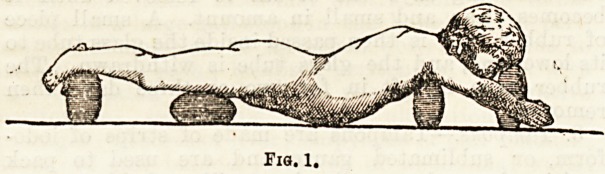


**Fig. 2. f2:**
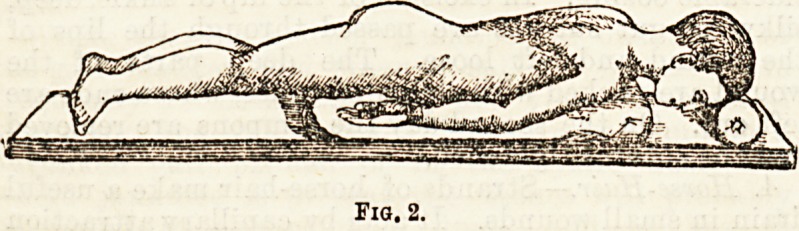


**Fig. 3. f3:**
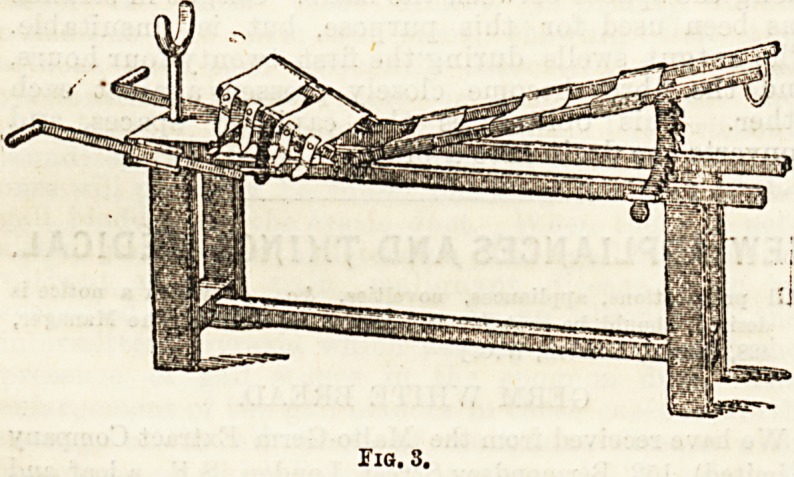


**Fig. 4. f4:**
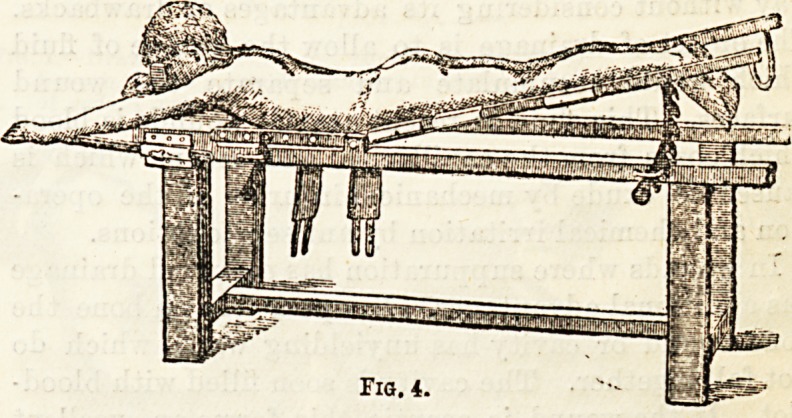


**Fig. 5. Fig. 6. f5:**